# Cerebral haemodynamics and carbon dioxide reactivity during sepsis syndrome

**DOI:** 10.1186/cc6185

**Published:** 2007-11-28

**Authors:** Christof Thees, Markus Kaiser, Martin Scholz, Alexander Semmler, Michael T Heneka, Georg Baumgarten, Andreas Hoeft, Christian Putensen

**Affiliations:** 1 Department of Anaesthesiology and Intensive Care Medicine, University of Bonn, 53105 Bonn, Germany; 2 Department of Neurology, University of Bonn, 53105 Bonn, Germany; 3 Department of Neurology, University of Bonn, 53105 Bonn, Germany; 4 Department of Anaesthesiology and Intensive Care Medicine, University of Bonn, 53105 Bonn, Germany; 5 Department of Anaesthesiology and Intensive Care Medicine, University of Bonn, 53105 Bonn, Germany

## Abstract

**Background:**

Most patients with sepsis develop potentially irreversible cerebral dysfunctions. It is yet not clear whether cerebral haemodynamics are altered in these sepsis patients at all, and to what extent. We hypothesized that cerebral haemodynamics and carbon dioxide reactivity would be impaired in patients with sepsis syndrome and pathological electroencephalogram patterns.

**Methods:**

After approval of the institutional ethics committee, 10 mechanically ventilated patients with sepsis syndrome and pathological electroencephalogram patterns underwent measurements of cerebral blood flow and jugular venous oxygen saturation before and after reduction of the arterial carbon dioxide partial pressure by 0.93 ± 0.7 kPa iu by ypervent ilation. The cerebral capillary closing pressure was determined from transcranial Doppler measurements of the arterial blood flow of the middle cerebral artery and the arterial pressure curve. A *t *test for matched pairs was used for statistical analysis (*P *< 0.05).

**Results:**

During stable mean arterial pressure and cardiac index, reduction of the arterial carbon dioxide partial pressure led to a significant increase of the capillary closing pressure from 25 ± 11 mmHg to 39 ± 15 mmHg (*P *< 0.001), with a consecutive decrease of blood flow velocity in the middle cerebral artery of 21.8 ± 4.8%/kPa (*P *< 0.001), of cerebral blood flow from 64 ± 29 ml/100 g/min to 39 ± 15 ml/100 g/min (*P *< 0.001) and of jugular venous oxygen saturation from 75 ± 8% to 67 ± 14% (*P *< 0.01).

**Conclusion:**

In contrast to other experimental and clinical data, we observed no pathological findings in the investigated parameters of cerebral perfusion and oxygenation.

## Background

Up to 71% of patients with sepsis develop potentially irreversible cerebral dysfunctions [1,2]. This sepsis-induced encephalopathy causes alteration of the mental state, ranging from mild disorientation or lethargy to coma and obtundation, and is commonly associated with abnormal electroencephalogram (EEG) patterns [2,3]. Several clinical investigations have demonstrated that sepsis-induced encephalopathy is an early sign of infection and may contribute to increased morbidity and mortality in septic patients [1,4].

Sepsis, the inflammatory response to infection, in critically ill patients provokes severe systemic haemodynamic disturbance, characterized by a high cardiac output despite evidence of myocardial dysfunction, low systemic vascular resistance, hypotension and regional blood flow redistribution resulting in tissue hypoperfusion. Scarce clinical data [5,6] and experimental data [7] show profound changes in cerebral blood flow associated with impaired carbon dioxide reactivity in severe sepsis and septic shock. Whether alterations of systemic or cerebral circulation might play a role in sepsis-induced encephalopathy, however, has not yet been determined.

In most of the former studies concerning cerebral haemodynamics during sepsis syndrome, only a few aspects of cerebral circulation had been investigated. We therefore tried to investigate simultaneously various parameters to obtain a more broad survey of cerebral perfusion and oxygenation in patients with sepsis syndrome showing abnormal EEG patterns.

## Materials and methods

In accordance with the Helsinki Declaration and after approval by the Bonn University ethics committee, 10 mechanically ventilated patients were studied in whom sepsis had been established for >48 hours. Informed consent was obtained from the patients or from their next of kin. The 1992 criteria of the American College of Chest Physicians and the Society of Critical Care Medicine Consensus Conference Committee were used to define sepsis [8]. Patients with a history of neurological disease and those with unstable cardiopulmonary function were not included in the study. The Multiple Organ Dysfunction Score [9] and the Acute Physiology and Chronic Health Evaluation II score [10] were assessed for the patients at inclusion in the study.

### Cardiovascular measurements

The heart rate was obtained from the electrocardiogram. The systemic mean blood pressure (MAP), the central venous pressure and the pulmonary artery pressure were transduced (Combitrans; Braun AG, Melsungen, Germany) and recorded (CS/3; Datex-Engström, Helsinki, Finland). The cardiac output was continuously estimated with the thermal dilution technique (Vigilance; Baxter Edwards Critical-Care, Irvine, CA, USA). Standard formulae were used to calculate the cardiac index (CI) and the systemic vascular resistance index.

### Cerebral circulation measurements

The blood flow velocity in the middle cerebral artery (V_MCA_) was measured by means of a 2 MHz transcranial Doppler probe (Multidop T; DWL, Singen, Germany). The Doppler probe was fixed to the patient's head using a specially designed holder apparatus (DWL) to ensure a constant angle of insonation during the study period. Transcranial Doppler adjustments of the depth, sample volume, gain, and power were kept constant during the investigation. Data for the arterial pressure and for the V_MCA _were stored simultaneously via analogue/digital converters with a sample rate of 114 Hz using the integrated hard disk of the transcranial Doppler device. Digital signals were then processed offline using a self-developed software (author MS). The cerebral capillary closing pressure (CCP) was calculated by heart-beat-to-heart-beat analysis from the zero-flow velocity pressure as extrapolated by regression analysis of arterial pressure/V_MCA _plots [11]. Since the arterial pressure and V_MCA _are dynamic values that fluctuate from beat to beat (for example, because of ventilation), CCP calculations had been averaged over a period of two respiratory cycles.

Transcerebral and transpulmonary double-indicator dilution methods were used to estimate the cerebral blood flow (CBF), cardiac output and intrathoracic blood volume as described previously [12,13]. Briefly, 25 mg indocyanine green dye (Becton Dickinson, Cockeysville, MD, USA) dissolved in 40 ml iced 5% glucose solution was used as a double-indicator and was injected into the right atrium via the central venous line. Dilution curves for the dye and the temperature were recorded simultaneously with the thermistor-tipped fibre-optic catheters (Pulsiocath PV 2024; Pulsion Medical Systems, München, Germany) in the aorta (30 cm catheter inserted in the femoral artery) and in the jugular bulb. All measurements were carried out from the, sonographically controlled, dominant (right) internal jugular vein. The CBF was calculated from the mean transit time of the first pass of the thermal and dye indicators with a computer (COLD-Z-021; Pulsion Medical Systems).

The cerebral metabolic rate of oxygen was calculated as the CBF multiplied by the arterial concentration of oxygen value minus the jugular venous concentration of oxygen value

### Electroencephalogram recordings

An EEG was recorded from each patient before the measurements. EEG recordings followed a standardized protocol on an analogue eight-channel recorder (Schwarzer GmbH, München, Germany) system with silver/silver chloride bridge electrodes placed according to the international 10–20 system. Examination was composed of recordings with two unipolar montages with the ipsilateral ear or the vertex electrode as reference, with two bipolar montages (longitudinal, transverse), and with a unipolar topo-selective and a unipolar Goldmann common reference montage. All EEG reports were analysed by a blinded EEG board-certified physician. EEG reporting was based on the EEG classification by Lüders and Noachter [14].

### Gas analysis

Arterial and jugular venous bulb blood gases and the pH were determined immediately after sampling with standard blood gas electrodes (ABL 620; Radiometer, Copenhagen, Denmark). The oxygen saturation and haemoglobin in each sample were analysed using spectrophotometry (OSM 3; Radiometer). The end-tidal expired carbon dioxide (ETCO_2_) was continuously measured (CS/3; Datex-Engström).

### Protocol

After inclusion in the study, all patients remained supine with a head-up position of 15°C. Adequate fluid supply was ensured with infusion of lactated Ringer's solution to achieve an intrathoracic blood volume index (ITBVI) between 900 and 1,000 ml/m^2^. Albumin 20% solution was given to maintain serum albumin concentrations above 2.0 g/dl, and packed red blood cells were administered to achieve haemoglobin of at least 10 g/dl. Dobutamine was infused when the CI fell below 3.5 l/min/m^2 ^despite fluid replacement, to achieve a CI between 3.5 and 4.5 l/min/m^2^. Norepinephrine infusion was added if the MAP was below 70 mmHg, to restore the MAP between 70 and 95 mmHg. Continuous infusion of sufentanil and propofol were titrated as clinically required to achieve a Ramsay sedation score of 3 [15]. Fluid replacement and infusion of all drugs then remained unchanged throughout the study.

Pressure-limited ventilatory support was provided with a standard ventilator (Evita; Dräger, Lübeck, Germany). The positive end-expiratory pressure and the pressure levels were adjusted to a tidal volume of 6 ml/kg and maximum lung compliance. The ventilator rate was set to maintain an arterial carbon dioxide partial pressure (P_a_CO_2_) between 5.3 and 6.6 kPa, and the inspiratory oxygen fraction was set to maintain an arterial oxygen partial pressure above 12 kPa. After baseline measurements were performed under normoventilation, the ventilatory rate was increased to result in a decrease in ETCO_2 _of 1.33 kPa (according to 10 mmHg). Changes of the blood gas status were controlled simultaneously by arterial blood gas analysis. Measurements and data collection were performed during stable steady-state conditions confirmed by constancy (± 5%) of the expiratory minute ventilation, the arterial oxygen saturation, the ETCO_2_, the MAP, and the CI for at least 40 minutes.

Three days after the cessation of continuous analgesia, sedation and extubation, the patients were neurologically examined each day by a certified neurologist.

For comparison, EEGs were recorded in 10 critically ill control patients without sepsis and systemic inflammatory response syndrome administered with a continuous infusion of sufentanil and propofol as clinically required to achieve a Ramsay sedation score of 3. All patients had been treated on our intensive care unit because of respiratory insufficiency after thoracic surgery. An absence of systemic inflammatory response syndrome was assured by the 1992 criteria of the American College of Chest Physicians and the Society of Critical Care Medicine Consensus Conference Committee [8].

### Statistical analysis

Results are expressed as the mean ± standard deviation. Differences between measurements were analysed by *t *test for matched pairs. Stepwise regression analysis was performed to analyse the relationship between carbon dioxide reagibility of the V_MCA_, CCP, CBF and jugular venous oxygen saturation (S_j_O_2_) and the age of the patients, the Acute Physiologic and Chronic Health Evaluation II score, the Multiple Organ Dysfunction Score, the body temperature, the arterial blood gas pH, the MAP, the CI, the systemic vascular resistance index and the ITBVI.

Between-group differences of pathology grades of the EEG recordings following the classification of Lüders and Noachter [14] were analysed with Student's *t *test. Differences were considered statistically significant if *P *< 0.05.

Statistical analysis was performed using STATISTICA 6.0 software (StatSoft Inc., Tulsa, OK, USA).

## Results

The patients' demographic and clinical data are summarized in Table 1. The mean Acute Physiologic and Chronic Health Evaluation II score was 31.2 ± 6.9, and the mean Multiple Organ Dysfunction Score was 13.8 ± 4.3.

**Table 1:** Patient demographic data at the timepoint of investigation

Patient	Age (years), gender	Underlying disease	APACHE II score	MODS	Day of investigation	CCT	Survival
1	32, male	Bacterial pneumonia following lung contusion	26	14	5	+	+
2	74, male	Necrotizing pancreatitis, secondary bacterial peritonitis	43	22	9	+	-
3	68, female	Necrotizing fasciitis	37	21	3	+	-
4	3, female	Bacterial pneumonia	23	10	5	-	+
5	28, male	Bacterial pneumonia following lung contusion	23	12	4	+	+
6	62, female	Perforated diverticulitis bacterial peritonitis	33	14	7	-	+
7	60, male	Bacterial pneumonia, secondary pleural empyema	26	10	3	+	+
8	46, male	Necrotizing pancreatitis, bacterial peritonitis	34	12	5	+	+
9	34, female	Necrotizing fasciitis	29	10	3	-	+
10	42, male	Necrotizing pancreatitis, secondary bacterial peritonitis	38	13	8	+	+
Mean ± standard deviation	48.5 ± 16.3		31.2 ± 6.9	13.8 ± 4.3			

APACHE II, Acute Physiology and Chronic Health Evaluation II score; MODS, Multiple Organ Dysfunction Score; day, day of investigation after onset of sepsis syndrome; CCT, cerebral computer tomography.

Ventilatory variables and ventilator settings are presented in Table 2. Mechanical ventilation with a positive end-expiratory pressure of 17 ± 3 mbar, an upper airway pressure limit of 27 ± 3 mbar, and an inspiratory oxygen fraction of 0.5 ± 0.22 resulted in a tidal volume of 439 ± 122 ml and an arterial oxygen partial pressure of 14.2 ± 3.2 kPa. When the ventilatory rate was set from 20 ± 3/min to 26 ± 3/min to achieve a reduction of ETCO_2 _of 1.33 kPa, the expiratory minute ventilation increased (*P *< 0.05) and the P_a_CO_2 _decreased from 5.85 ± 1.06 kPa to 4.92 ± 1.06 kPa (*P *< 0.01). The MAP, positive end-expiratory pressure, and tidal volume remained essentially constant throughout the intervention.

**Table 2:** Ventilatory variables and ventilator settings before and after reduction of the arterial carbon dioxide partial pressure (P_a_CO_2_)

	Baseline	Decreased P_a_CO_2_
Relative risk (1/min)	20 ± 3	26 ± 3*
Tidal volume (ml)	439 ± 122	422 ± 146
Expiratory minute ventilation (l/min)	9.3 ± 2.6	13.3 ± 3.7*
Airway pressure (mbar)	21 ± 4	21 ± 4
Positive end-expiratory pressure (mbar)	17 ± 3	17 ± 3
Arterial oxygen partial pressure (kPa)	14.2 ± 3.2	13.8 ± 3.6
Arterial oxygen saturation (%)	97 ± 1	97 ± 1
P_a_CO_2 _(kPa)	5.85 ± 1.06	4.92 ± 1.06*
pH	7.38 ± 0.1	7.41 ± 0.1*

*P < 0.05, matched pairs *t *test, *n *= 10.

Changes in cardiovascular variables are presented in Table 3. Continuous infusion of 0.28 ± 0.22 μg/kg/min norepinephrine and 7.9 ± 4.7 μg/kg/min dobutamine was necessary to achieve a CI of 4.2 ± 1.8 l/min/m^2 ^and a MAP of 89 ± 15 mmHg. Hyperventilation did not affect cardiovascular function.

**Table 3:** Systemic circulatory variables before and after reduction of the arterial carbon dioxide partial pressure (P_a_CO_2_)

	Baseline	Decreased P_a_CO_2_
Heart rate (1/min)	104 ± 18	108 ± 19
Mean arterial pressure (mmHg)	89 ± 15	87 ± 16
Central venous pressure (mmHg)	15 ± 5	15 ± 6
Pulmonary arterial pressure (mmHg)	26 ± 5	25 ± 5
Intrathoracic blood volume index (ml/m^2^)	1032 ± 202	988 ± 231
Systemic vascular resistance index (dyn/s/cm^-5^/m^2^)	899 ± 382	874 ± 358
Cardiac index (l/min/m^2^)	4.2 ± 1.8	4.1 ± 1.9

There were no significant differences between baseline values and reduction of the P_a_CO_2 _(*P *< 0.05, matched pairs *t *test), *n *= 10.

**Figure 1:**
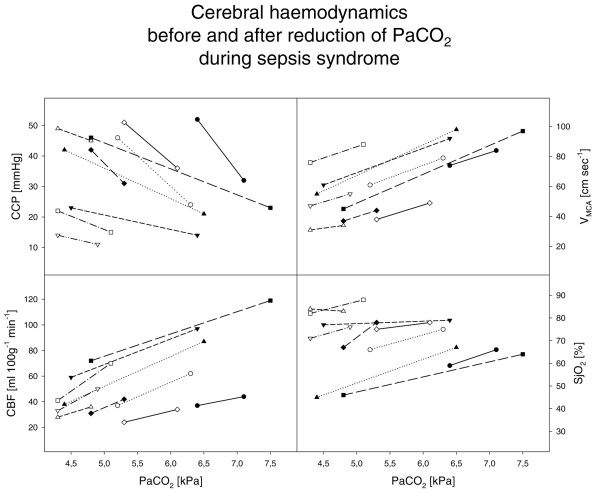
Changes in cerebral circulatory variables. Cerebral blood flow (CBF), blood flow velocity in the middle cerebral artery (V_MCA_), cerebral critical closing pressure (CCP) and venous oxygen saturation in the jugular bulb (S_j_O_2_) in 10 patients during sepsis syndrome before and after reduction of the arterial carbon dioxide partial pressure (P_a_CO_2_).

Changes in cerebral circulatory variables are shown in Table 4 and Figure 1. Hyperventilation with a reduction of the P_a_CO_2 _of 0.93 ± 0.7 kPa (range, 0.5–2.7 kPa) resulted in a decrease in the V_MCA _from 72 ± 25 cm/s to 59 ± 22 cm/s (*P *< 0.001). The mean decrease in the V_MCA _was 21.8 ± 4.8%/kPa, with a range from 17 to 32%/kPa. While the CCP increased from 25 ± 11 mmHg to 39 ± 15 mmHg (*P *< 0.001), the CBF decreased from 64 ± 29 ml/100 g/min to 39 ± 15 ml/100 g/min (*P *< 0.001) and the mean S_j_O_2 _from 75 ± 8% to 67 ± 14% (*P *< 0.01). The cerebral metabolic rate of oxygen was 1.9 ± 0.8 ml/100 g/min and did not change significantly during hyperventilation.

**Table 4:** Variables of cerebral circulation and oxygenation before and after reduction of the arterial carbon dioxide partial pressure (P_a_CO_2_) by 0.93 kPa

	Baseline	Decreased P_a_CO_2_
Cerebral blood flow (ml/100 g/min)	64 ± 29	39 ± 15**
Blood flow velocity in the middle cerebral artery (cm/s)	72 ± 25	59 ± 22**
Cerebral critical closing pressure (mmHg)	25 ± 11	39 ± 15**
Physiological effective cerebral perfusion pressure^a ^(mmHg)	65 ± 16	48 ± 17**
Cerebral metabolic rate of oxygen (ml/100 g/min)	1.9 ± 0.8	1.9 ± 0.9
Venous oxygen saturation in the jugular bulb (%)	75 ± 8	67 ± 14*

^a^Mean arterial pressure minus cerebral critical closing pressure. *P < 0.01 and **P < 0.001, matched pairs *t *test, *n *= 10.

None of the studied factors (age of the patients, Acute Physiologic and Chronic Health Evaluation II score, Multiple Organ Dysfunction Score, body temperature, arterial blood gas pH, MAP, CI, systemic vascular resistance index, and ITBVI) had any significant association with cerebrovascular carbon dioxide reactivity.

During the stay on the intensive care unit, cerebral computer tomography scans had been carried out in seven of the 10 patients after our measurements (Table 1). None of these patients showed pathological findings.

**Figure 2:**
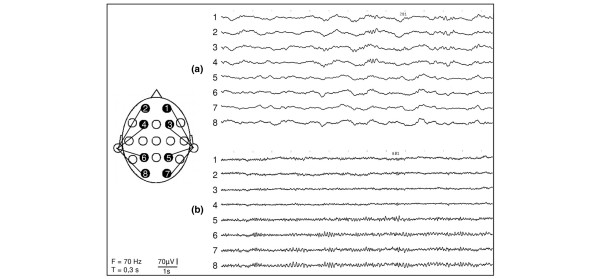
Representative Electroencephalogram samples of sepsis patients (a) and control patients (b). (F: filter setting, T: paper transport)

The EEG recordings in the septic patients showed slowing of the background rhythm, as well as intermittent or continuous regional slowing and epileptiform potentials, indicating a severe brain dysfunction during sepsis. The control patients showed no or only mild EEG abnormalities. The average EEG pathology grade [14] was 1.9 in the sepsis group and was 0.5 in the control group (*P *< 0.01). Figure 2 shows representative EEG samples in a unipolar montage with the ipsilateral ear as reference from (a) a patient with sepsis syndrome and (b) a control patient. (a) Generalized slowing of the EEG rhythm. (b) Normal EEG recording in the nonseptic control group.

Nine of the 10 patients came to our intensive care unit in deep anaesthesia after surgical intervention. No neurological conspicuousness had been found for the patients in the initial exploration by the anaesthesiologist or surgeon, except for a slight drowsiness in three cases according to Glasgow Coma Scale 14. Eight of the 10 patients survived. Two patients died due to multiple organ failure. All surviving patients showed pathological findings on clinical neurological exploration during the first 5 days after extubation: 3 days after cessation of continuous analgesia, sedation and extubation, their consciousness was severely reduced (mean ± standard deviation Glasgow Coma Score, 12 ± 1; range, 11–14) without application of sedation. While none of the patients were oriented in regard to time and location, five were disoriented in regard to person. Four of the patients suffered from psychotic symptoms.

## Discussion

In the present investigation a reduction of the P_a_CO_2 _led to a significant increase in the CCP with a consecutive decrease in the V_MCA_, CBF and S_j_O_2_. Despite neurological disorder and pathological EEG patterns, none of the recorded variables of cerebral circulation was pathological in the 10 investigated patients.

Experimental and clinical investigations demonstrated disturbed cerebral perfusion during sepsis or septic shock. The question of whether the cerebral carbon dioxide vasomotor reactivity is concomitantly impaired remained unclear. In a previous animal experimental study [7], cerebral vascular reactivity was reduced. Clinical data, however, are contradictory. Matta and Stow reported only a slightly altered cerebral carbon dioxide reactivity, but their conclusions were limited to the early stages of sepsis in their group of investigated patients [16]. Moller and colleagues investigated the CBF after an intravenous bolus of endotoxin in healthy volunteers [17]. During endotoxinaemia they observed a decrease in CBF during a simultaneous reduction of the P_a_CO_2_. The authors concluded that endotoxinaemia does not alter cerebral perfusion, and they explained the reduced CBF by acute hypocapnia caused by hyperventilation of their spontaneous breathing patients, indicating intact cerebral carbon dioxide reactivity. Conversely, in clinical trials using transcranial Doppler, Terborg and colleagues [5] and Bowie and colleagues [6] observed significantly impaired cerebral carbon dioxide reactivity of the V_MCA _during sepsis syndrome.

The intention of the present investigation was to gain a broader overview of the cerebral haemodynamics during sepsis syndrome by recording simultaneously different parameters of the cerebral circulation and oxygenation before and after reduction of P_a_CO_2_.

In agreement with Panerai [18], who emphasized the necessity of CCP monitoring to obtain more accurate estimates of cerebrovascular resistance changes, we recorded the CCP using transcranial Doppler sonography as previously described [11]. This major component of the effective organ downstream pressure [19] is determined besides tissue pressure by venous backpressure, and especially by vasomotor tone [20]. During constant tissue pressure (intracranial pressure) and constant venous backpressure, changes in the CCP predominantly reflect changes in vasomotor tone. The CCP could therefore be used as a direct measure of carbon dioxide reactivity in our investigation. The intrathoracic pressure and central venous pressure did not change during the measurements. Beyond that, it can be presumed that the intracranial pressure did not change or rather decreased during P_a_CO_2 _reduction. This would have caused a more modest increase in the CCP, and therefore an underestimation of cerebral vasomotor reactivity.

A control of our measurements in the same patients after recovery from sepsis was not feasible because of different difficulties: the lack of cooperation of the surviving patients suffering from psychotic symptoms, the difficulty of proper CBF measurements caused by artefacts during spontaneous breathing, and the lack of clinical indication of jugular bulb oxymetry after recovery from septic shock. We therefore had to compare our results with investigations focusing on the same parameters of the cerebral circulation in patients without severe inflammatory response syndrome or sepsis. In our patients, the mean decrease in the P_a_CO_2 _by 0.93 kPa led to a mean increase in the CCP of 14 mmHg. In patients recovering from head injury, Weyland and colleagues [21] recorded a mean change in CCP of only 6 mmHg during variation of the P_a_CO_2 _by about 1.06 kPa. This difference in CCP after varying the P_a_CO_2 _was in a distinctly smaller range than that observed in our septic patients. Of course, a comparison with these results is rather difficult because it is not improbable that, during recovery after brain injury, the cerebral perfusion is still disturbed. Nevertheless, cerebral carbon dioxide reactivity in our investigation seems to be normal rather than reduced. This conclusion is supported by the simultaneous recorded V_MCA _and CBF values.

As expected, the increase in the CCP, and thus cerebral vasomotor tone, was accompanied by a decreased CBF, which is reflected in a reduced V_MCA_. In contrast to the observations of two previous investigations [5,6], the decrease in the V_MCA _(21.8 ± 4.8%/kPa) was in a normal range [6,22]. Terborg and colleagues investigated septic patients with neurological illness that may have impaired cerebrovascular reactivity – a possible explanation for the differing results[5]. The patients investigated by Bowie and colleagues [6] seem to be quite comparable with those of our study. The data of systemical circulation (MAP and CI) are quite similar except for a distinctly higher mean systemic vascular resistance index. The haemodynamic management of septic patients in our department is ITBVI oriented, aiming at rather high intravascular volume for optimized organ perfusion resulting in lower vascular resistance during sufficient MAP. Effects of systemic haemodynamics on cerebral circulation (for example, CI during septic shock) have been demonstrated [23]. Nevertheless, effects of a potential higher ITBVI on cerebral carbon dioxide reactivity remain speculative.

The hyperventilation of the patients in our study was ETCO_2 _oriented. An end-tidal partial pressure reduction of 1.33 kPa resulted in a deviant mean decrease in the P_a_CO_2 _of 0.93 ± 0.7 kPa, with a wide range of 0.5–2.7 kPa reflecting the disturbance of pulmonary function and perfusion in the septic patients. The calculation of cerebral carbon dioxide reactivity by Bowie and colleagues based on the ETCO_2 _may also contribute to the different results [6].

Global CBF was measured using a transcerebral double-indicator dilution technique. The few validation studies have shown sufficient agreement with an inert-gas technique using argon in patients with normal cerebrovascular function [12], whereas overestimation of cerebral perfusion was observed in patients with brain injury or subarachnoid haemorrhage [24]. The reproducibility was fairly good and comparable with other methods for CBF measurement [25]. Although not widely used, a transcerebral double-indicator dilution technique seemed suitable in particular in our investigation because it allows easy bedside measurements with simultaneous recording of various other parameters.

Wietasch and colleagues [12] and Mielck and colleagues [13] varied the P_a_CO_2 _in patients scheduled for coronary bypass surgery. They recorded the CBF by the same transcerebral double-indicator dilution technique used in our investigation. In both studies, during normocapnia the CBF (40 ± 6 ml/100 g/min and 39 ± 14 ml/100 g/min, respectively) was lower than in the septic patients of our investigation (64 ± 29 ml/100 g/min). Variations of the P_a_CO_2 _by 1.46 kPa led to changes in the CBF to about 22 and 24 ml/100 g/min, respectively. Compared with these non-septic patients, the CBF decrease in our group of patients was in the same range – although the mean reduction of the P_a_CO_2 _was only 0.93 kPa. Investigations on the regional CBF using ^133^Xe methods [26] also showed a more slight reaction to changes in the P_a_CO_2 _(4% regional CBF per 0.13 kPa P_a_CO_2_). The effects of cerebral carbon dioxide reactivity on global cerebral perfusion are therefore rather more distinct in our investigation despite the fact that the patients suffered from sepsis syndrome. A consecutive decrease in the S_j_O_2 _from 75% to 67% reflects this reduction of the cerebral perfusion.

We found pathological activity in the EEG for all septic patients, with significant difference from the nonseptic control patients that cannot be explained by sedation. Both patient groups had comparable sedation as clinically required to achieve a Ramsay sedation score of 3, sufficient for toleration of airway pressure release ventilation respirator therapy including spontaneous breathing. Although the EEG changes are not specific for septic encephalopathy, at least an influence of sepsis must be postulated. Also nonspecific were the pathological findings in clinical neurological exploration of the eight surviving septic patients. Effects of sedation are conceivable. Three days after the cessation of sedation, however, this seems unlikely because sedation had been performed as Ramsay score oriented to avoid accumulation using the short-reacting propofol.

## Conclusion

In contrast to the experimental and clinical data of Rudinsky and colleagues [7], of Terborg and colleagues [5] and of Bowie and colleagues [6], carbon dioxide reactivity seemed not to be impaired during sepsis syndrome in our patients. None of the recorded parameters of cerebral perfusion and oxygenation seemed causative for the observed pathological findings in EEG and clinical neurological exploration at the time point of investigation. Cerebral autoregulation was not investigated. Nevertheless, the patients had been haemodynamically stabilized to each time point of their stay in our hospital. Global cerebral hypoperfusion caused by insufficient CPP during septic shock as observed by Wijdicks and Stevens [27] can be excluded as a reason for encephalopathic symptoms. Although cerebral computer tomography scans in seven of the 10 patients showed no pathological findings, disturbance of regional cerebral perfusion cannot be excluded. Further investigation is therefore needed for a definite elucidation of the role of cerebral haemodynamics in the origin of septic encephalopathy.

## Key messages

• Cerebral haemodynamics and carbon dioxide reagibility were investigated in 10 mechanically ventilated patients with sepsis syndrome.

• Reduction of P_a_CO_2 _caused a significant increase in the cerebral capillary closing pressure, with a consecutive decrease in the blood flow velocity of the middle cerebral artery, the global cerebral blood flow and the jugular venous oxygen saturation.

• No pathological findings have been observed.

## Abbreviations

CBF = cerebral blood flow; CCP = capillary closing pressure; CI = cardiac index; EEG = electroencephalogram; ETCO_2 _= end-tidal carbon dioxide partial pressure; ITBVI = intrathoracic blood volume index; MAP = mean arterial pressure; P_a_CO_2 _= arterial carbon dioxide partial pressure; S_j_O_2 _= jugular venous oxygen saturation; V_MCA _= blood flow velocity in the middle cerebral artery.

## Competing interests

The authors declare that they have no competing interests.

## Authors' contributions

CT made substantial contributions to the conception and design of the study and to acquisition, analysis and interpretation of the data, and prepared the manuscript. MK made substantial contributions to the acquisition, analysis and interpretation of data and participated in the preparation of the manuscript. MS made substantial contributions to the analysis and interpretation of data, especially development of the software for measurement of the cerebral capillary closing pressure. AS made substantial contributions to the acquisition, analysis and interpretation of data, especially the EEG recordings, performed the statistical analysis and participated in the preparation of the manuscript. MTH made substantial contributions to the conception and design of the study, and to analysis and interpretation of the data, especially the EEG recordings. GB made substantial contributions to the acquisition and analysis of data. AH made substantial contributions to the conception and design of the study and has revised the manuscript for important intellectual content. CP made substantial contributions to the conception and design of the study, was involved in the preparation of the manuscript, revising it for important intellectual content, and has given final approval of the version published.
